# The Role of 1.5 Tesla MRI and Anesthetic Regimen Concerning Cardiac Analysis in Mice with Cardiomyopathy

**DOI:** 10.1371/journal.pone.0094615

**Published:** 2014-04-18

**Authors:** Ulrich Grabmaier, Hans D. Theiss, Alexandra Keithahn, Julia Kreiner, Christoph Brenner, Bruno Huber, Christine von der Helm, Lisa Gross, Karin Klingel, Wolfgang-M. Franz, Stefan Brunner

**Affiliations:** 1 Department of Internal Medicine I, Ludwig-Maximilians-University, Campus Grosshadern, Munich, Germany; 2 Department of Nuclear Medicine, Klinikum Rechts der Isar, Munich, Germany; 3 Department of Molecular Pathology, University of Tübingen, Tübingen, Germany; 4 Department of Dermatology, Klinikum Augsburg, Augsburg, Germany; Massachusetts General Hospital, United States of America

## Abstract

Accurate assessment of left ventricular function in rodent models is essential for the evaluation of new therapeutic approaches for cardiac diseases. In our study, we provide new insights regarding the role of a 1.5 Tesla (T) magnetic resonance imaging (MRI) device and different anesthetic regimens on data validity. As dedicated small animal MRI and echocardiographic devices are not broadly available, we evaluated whether monitoring cardiac function in small rodents with a clinical 1.5 T MRI device is feasible. On a clinical electrocardiogram (ECG) synchronized 1.5 T MRI scanner we therefore studied cardiac function parameters of mice with chronic virus-induced cardiomyopathy. Thus, reduced left ventricular ejection fraction (LVEF) could be verified compared to healthy controls. However, our results showed a high variability. First, anesthesia with medetomidine, midazolam and fentanyl (MMF) led to depressed cardiac function parameters and more variability than isoflurane gas inhalation anesthesia, especially at high concentrations. Furthermore, calculation of an average ejection fraction value from sequenced scans significantly reduced the variance of the results. To sum up, we introduce the clinical 1.5 T MRI device as a new tool for effective analysis of left ventricular function in mice with cardiomyopathy. Besides, we suggest isoflurane gas inhalation anesthesia at high concentrations for variance reduction and recommend calculation of an average ejection fraction value from multiple sequenced MRI scans to provide valid data and a solid basis for further clinical testing.

## Introduction

Myocarditis is an inflammatory disease of the heart which is frequently caused by viral pathogens. Patients present with varying clinical symptoms ranging from mild dyspnea and benign cardiac arrhythmias to cardiogenic shock and sudden cardiac death [1]. In genetically susceptible individuals, viral infection of the heart can trigger ongoing myocardial inflammation subsequently leading to dilative cardiomyopathy and congestive heart failure [2]. In these patients, optimized medication often represents the only therapeutic option, though frequent progression to end stage heart failure and death cannot be avoided [2]–[4]. Hence, new therapeutic strategies targeting acute inflammation and/or preventing the onset of dilative cardiomyopathy are crucial. In the process of developing new therapeutic approaches, animal models play an essential role. Since left ventricular ejection fraction (LVEF) strongly predicts cardiovascular morbidity and mortality, it is often used for monitoring therapeutic success [5]–[8]. Several techniques, such as pressure-volume loops [9], echocardiography [10] or dedicated small animal MRI scanners [11] have been implemented for evaluation of left ventricular function in mice. However, 1.5 Tesla MRI scanners have not been used routinely for cardiac function monitoring in murine models of dilative cardiomyopathy (DCM) so far.

Against this background, the objective of this pilot study was providing the feasibility of cardiac function evaluation by magnetic resonance imaging with a clinical 1.5 T MR scanner in coxsackievirus B3 (CVB3) infected mice, a mouse model for viral myocarditis and dilative cardiomyopathy. We therefore assessed left ventricular function parameters in coxsackievirus B3 infected mice and compared them to healthy controls. In order to verify our results with a more standardized imaging technique, we compared our data to results obtained with a dedicated small animal echocardiographic device. Furthermore, we compared the anesthetic regimen used in the MRI study with different concentrations of isoflurane to assess the optimal anesthetic conditions for data acquisition.

## Materials and Methods

### Animal model

SWR/J (H-2^q^) male mice (The Jackson Laboratories, USA) were infected intraperitoneally (i.p.) with 1×10^5^ plaque forming units (pfu) of coxsackievirus B3 (CVB3) [12], which finally resulted in a phenotype of dilative cardiomyopathy after several weeks p.i. as described [13], [14].

Cardiac function of 22 infected mice (SWR/J) was assessed by cardiac magnetic resonance imaging (MRI) eight weeks after CVB3 infection. For comparison of LVEF, 7 healthy mice (SWR/J) were tested as well. For comparison of left ventricular volumes and cardiac output, 4 age matched mice were used respectively.

Healthy, 6–8 week old male BALB/c mice were used for evaluation of cardiac function by echocardiography. Animal care and all experimental procedures were performed in strict accordance to the Guide for the Care and Use of Laboratory Animals published by the US National Institutes of Health (NIH publication No. 85-23, revised 1996) and were approved by the local animal care and use committees of the district government of Baden-Württemberg.

### Cardiac magnetic resonance imaging

For reliable electrocardiographic (ECG) synchronization and high resolution imaging, a dedicated small animal ECG device (1025-MR, SA Instruments Inc., Stony Brook, NY) and a microscopy coil (Philips Medical Systems, Best, NL), which is clinically used for finger imaging, were applied. All imaging procedures were performed on a clinical 1.5 T Philips Achieva MR scanner using a clinical gradient system (30 mT/m, 150 mT/m/ms). Animals (SWR/J) were anesthetized with fentanyl (10 µg/kg), medetomidine (1 mg/kg) and midazolam (2 mg/kg) (MMF) and imaged in prone position with the thorax positioned on top of the microscopy single loop surface coil (D = 2.3 mm). High-resolution MRI sequences for assessment of myocardial function and morphology were implemented. Cine MRI was performed with prospective ECG triggering using a spoiled gradient echo technique. Imaging parameters included repetition time/echo time (TR/TE) = 18 ms/6.5 ms, flip angle = 30°, averages = 1, field of view (FOV) = 35 mm, matrix = 128 resulting in a spatial resolution of 0.22×0.22×1 mm at a temporal resolution of 18 ms. Ejection fraction was determined by evaluation of end diastolic volumes (EDV) and end systolic volumes (ESV) in short axis view in seven slices with an in-house developed IDL 6.1 based software package. Calculation of left ventricular ejection fraction (in %) was conducted via the formula ((EDV-ESV)/EDV)*100.

### Echocardiographic imaging

**Figure 1:**
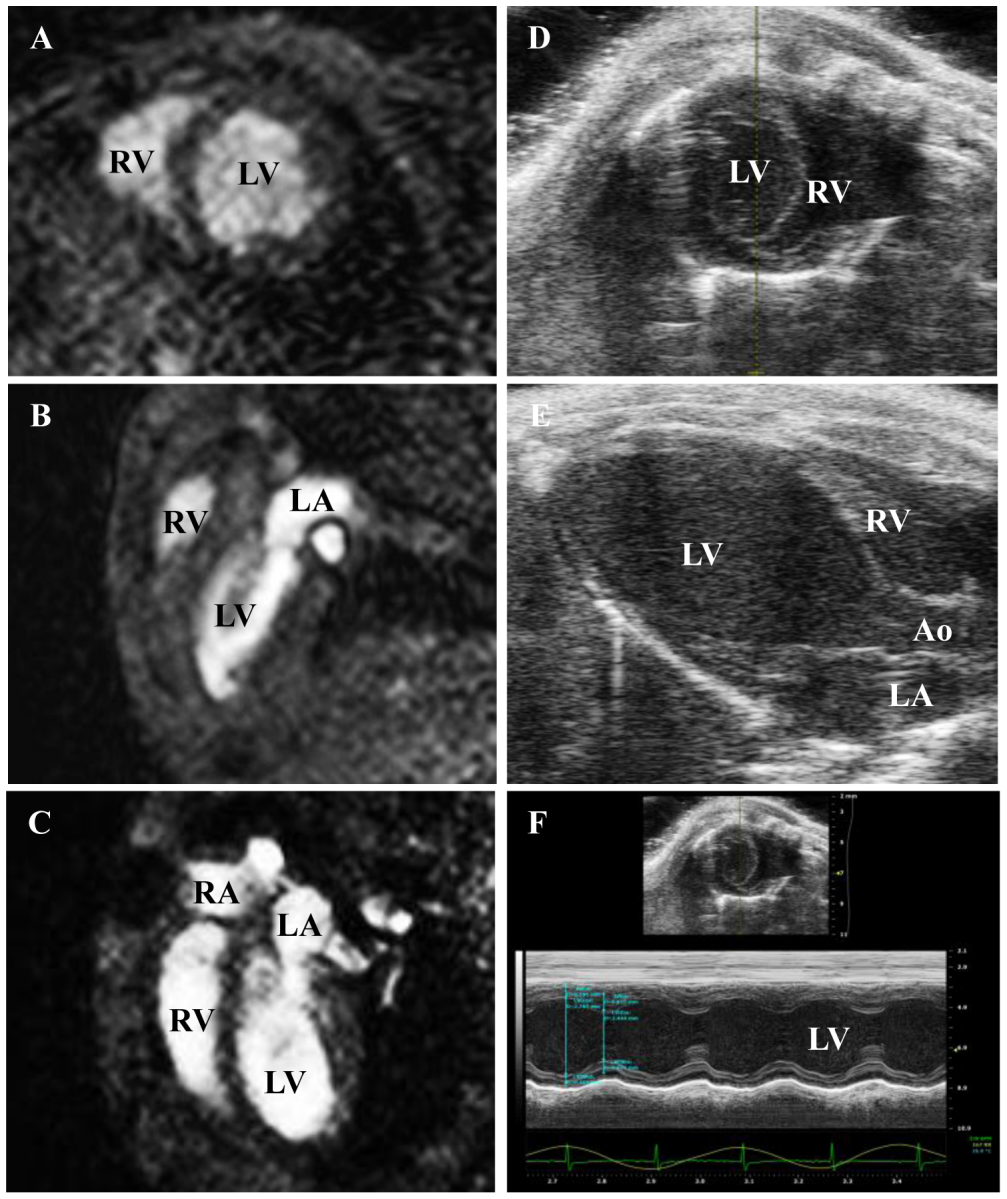
Representative slides of MRI and echocardiography-gained heart slides. Short axis view gained with A) MRI and D) echocardiography. Seven short axis slices were used for calculation of left ventricular volumes with MRI. B) Shows MRI-gained and E) echocardiography-gained long axis view. C) Shows 4-chamber view (MRI). F) Shows M-mode based calculation of LVEF (echocardiography). LV: left ventricle; RV: right ventricle; LA: left atrium; RA: right atrium; Ao: aorta.

Anesthesia of 6–8 week old, healthy male BALB/c mice (Charles River, Germany) was performed with 2% isoflurane. As ejection fractions of different mouse strains are comparable [11], [15]–[17], a mouse strain which is easily accessible (BALB/c) was used in this study. Once fully anesthetized, the mice were transferred to a heated platform (40°C) ensuring a stable body temperature of 34.5–35.5°C. Anesthesia was maintained with 1.75% or 3% isoflurane or intraperitoneal injection of 100 µl MMF. Transthoracic echocardiographic short-axis M-mode images (Fig. 1F) were obtained with a Vevo2100 imaging system (VisualSonics, Toronto, Canada) using a 40 MHz transducer. Parasternal long-axis M-mode was used to standardize transducer positioning for short-axis M-mode imaging. For evaluation of cardiac function, 3 consecutive cycles were measured and averaged as described previously [10], [18]. Left ventricular end diastolic (EDV) and systolic (ESV) volumes were calculated using the formulae 7*LVID;d^3^/(2.4+LVID;d) and 7*LVID;s^3^/(2.4+LVID;s). Calculation of left ventricular ejection fraction (in %) was conducted via the formula ((EDV-ESV)/EDV)*100. Cardiac index was defined as cardiac output divided by body weight as previously described [19].

### Statistical analysis

Data is shown as mean ± standard deviation (SD). Data was evaluated for normal distribution by the Anderson-Darling-Test. Comparison between two groups was performed using the unpaired t-test, if data was normally distributed. If values were significantly different from normal distribution, Mann-Whitney-U-test was used for statistical testing. Values of p<0.05 were considered statistically significant. Data were analysed using Microsoft Office 2010 and the statistical software PAST v2.17c [20]. Dot plot figures were designed with SPSS statistics 21.

## Results

### Evaluation of cardiac function using a clinical 1.5 T MRI device

In this model of cardiomyopathy, cardiac function of 22 CVB3-infected mice was assessed using cine MRI and results were matched with up to 7 healthy controls in order to evaluate the grade of systolic impairment. In figure 1A–C, representative MRI-gained slides in short axis, two-chamber and four-chamber view are shown. Table 1 shows end diastolic volume, end systolic volume, stroke volume (SV), left ventricular ejection fraction, cardiac output (CO), cardiac mass (mass) and heart rate (HR). LVEF was significantly reduced by 11% in coxsackievirus B3 infected mice compared to healthy controls. EDV of infected mice and healthy controls was not significantly different (44±11 µl vs. 52±12 µl, p = 0.26). There was no ESV difference between the two groups as well (26±7 µl in CVB3-infected mice and 27±7 µl in healthy mice, p = 0.79). SV of infected mice was decreased by ∼28% (18±5 µl in CVB3-infected mice and 25±5 µl in healthy mice, p = 0.06) and heart mass was comparable between groups (52±10 µl in CVB3-infected mice and 58±5 µl in healthy mice, p = 0.09). Finally, calculation of cardiac output (CO) revealed reduction of 38% in infected mice – this trend was almost statistically significant (3.8±1.4 ml/min in CVB3-infected mice and 6.1±1.6p ml/min in healthy mice, p = 0.06).

**Table 1:** Left ventricular function parameters of CVB3-infected mice compared to age matched healthy controls at baseline.

	Units	CVB3-infected	Healthy controls	P
EDV	µl	44±11	52±12	0.26
ESV	µl	26±7	27±8	0.79
SV	µl	18±5	25±5	0.06
LVEF	%	41.0±4.6	52.0±7.7	0.008
CO	ml/min	3.8±1.4	6.1±1.6	0.06
HR	/min	212±55	246±16	0.02
mass	mg	52±10	58±5	0.09

*Values are presented as mean and standard deviation, along with the corresponding p values.*

*(Student's t test).*

Our clinical 1.5 Tesla device included a microscopy coil which is clinically used for finger imaging and resulted in a lower signal-to-noise ratio in comparison to dedicated small animal 7 to 11.7 Tesla MRI devices combined with high-end gradient systems. In practice, this led to an increase of standard deviations. As shown in table 2, standard deviations for left ventricular volumes and cardiac function exceeded the values from indicated studies up to four times regardless of the mouse model used. These findings were consistent for diseased mice as well as for healthy controls. LVEF in our study (52.02%±7.72) was reduced by ∼20% compared to MRI data of healthy control mice (mean 62.4±3.1%) [11], [16], [17], [21].

**Table 2:** Comparison of MRI devices and results on cardiac function in small animals and in humans.

	Tesla	gradient	TR/TE in ms	Flip angle	FOV	spatial resolution	species	Model	n per group	Control groups	EF of controls in %	mean relative SD EDV/ESV/SV (%)	mean relative SD of LVEF (%)
**Grabmaier et al.**	1.5	30 mT/m	18/6.5	30°	35 mm	220×220×1000 µm	SWR/J	DCM	(4)7–22	y	52.0±7.7	23.5/26.5/23.6	13.1; 14.8*
**Nindl et al.**	9.4	400 mT/m	50.5/1.8	-	25×25 mm^2^	98×98×98 µm	BALB/c	DCM	6–7	n	62.8±2.0; 63.0±1.3	6.0/9.6/5.8	2.9; 2.6*
**Cochet et al.**	9.4	950 mT/m	4.3/1.1	20°	11×17×2 mm	133×133×133 µm	C57Bl/6	HCM	15	n	-	10.0/17.9/-	0.1
**Stuckey et al.**	11.7	-	4.6/1.4	17.5°	25.6×25.6	100×100×1000 µm	mdx mouse	mdx mouse	10–12	y	65±2; 60±2;	6.1/9.5/6.0	3.7
							model	model of			65±2; 60±3;		3.6*
							//	muscular			59±2		
							C57Blk10	dystrophy					
**Protti et al.**	7	-	-	-	-	-	C57Bl/6J	LAD ligation	-	n	65.6±4.3	20.5/27.3/28.0	6.6
**Klem et al.** #	1.5	-	-	-	-	-	human	ICM	100–906	n	-	-	34.4
**Gardner et al.** #	1.5	-	-	45°	35×35 cm	2.2×1.3×1.3 mm	human	ICM	30–47	y	-	-	15.4; 9.2*

# *= LVEF evaluation in human populations;*

*y = yes, n = no; SD = standard deviation;*

* = mean relative SD in % of LVEF of the control group only.

### Validation of LVEF-results by echocardiography (Vevo2100)

**Figure 2:**
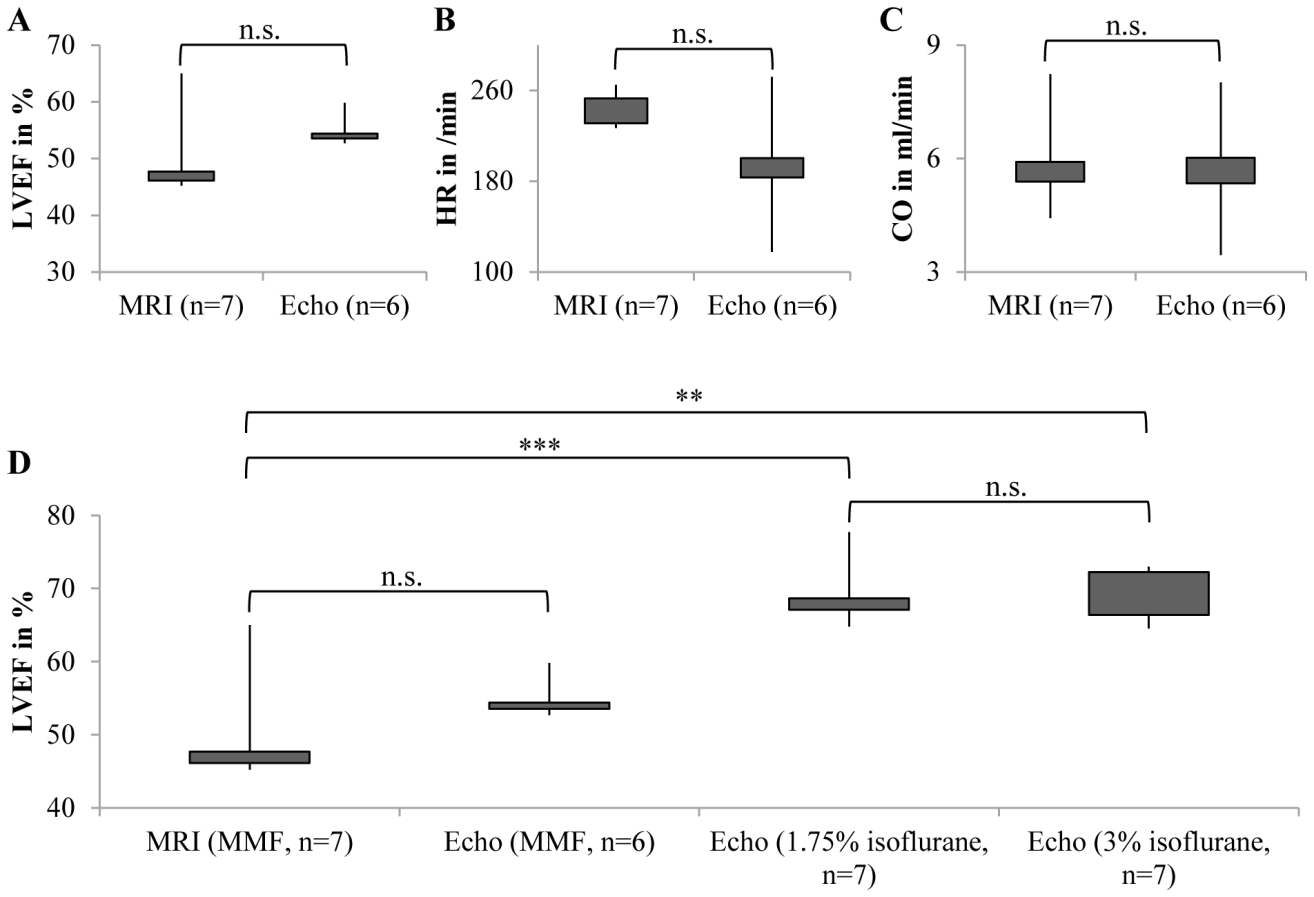
Echocardiography confirms values obtained by MRI. A) Echocardiography was able to reproduce the low LVEF values we obtained from MMF anesthetized mice investigated by MRI. B) Heart rates (HR) and C) cardiac output (CO) measured by our Vevo2100 system did not significantly differ from those in the MRI study. D) Anesthesia with isoflurane revealed the cardiodepressive effects of MMF. n.s.: not significant; **: p<0.01; ***: p<0.001.

As shown in table 2, LVEF of healthy control mice was reduced by ∼20% compared to results obtained from several indicated study groups. To evaluate whether the reduced ejection fraction was due to the chosen anesthetic regimen, we analyzed cardiac function of healthy mice with a dedicated small animal echocardiographic device as well. Figure 1D–F shows representative echo-images of short-axis view, parasternal long-axis view and M-mode of parasternal short-axis view. As shown in figure 2, evaluation of cardiac function with the echocardiographic device resulted in similar results for (A) ejection fraction (55.7±3.2% vs. 52.0±7.7%, p = 0.28), (B) heart rate (198±51/min vs. 246±16/min, p = 0.07) and cardiac output (6.1±1.7 ml/min vs. 6.1±1.6 ml/min, p = 0.96) when mice were anesthetized with the same regimen as used in the MRI study.

Isoflurane gas inhalation anesthesia of the same population revealed higher values for LVEF similar to the values obtained by other groups (table 2), indicating that the LVEF values obtained in our MRI study were due to the sedation protocol. Nevertheless, the standard deviation for LVEF was more than 2 times higher for MRI assessment of LVEF (relative SD 5.7% vs. 14.8%).

### Cardiodepressive effects of MMF

**Figure 3:**
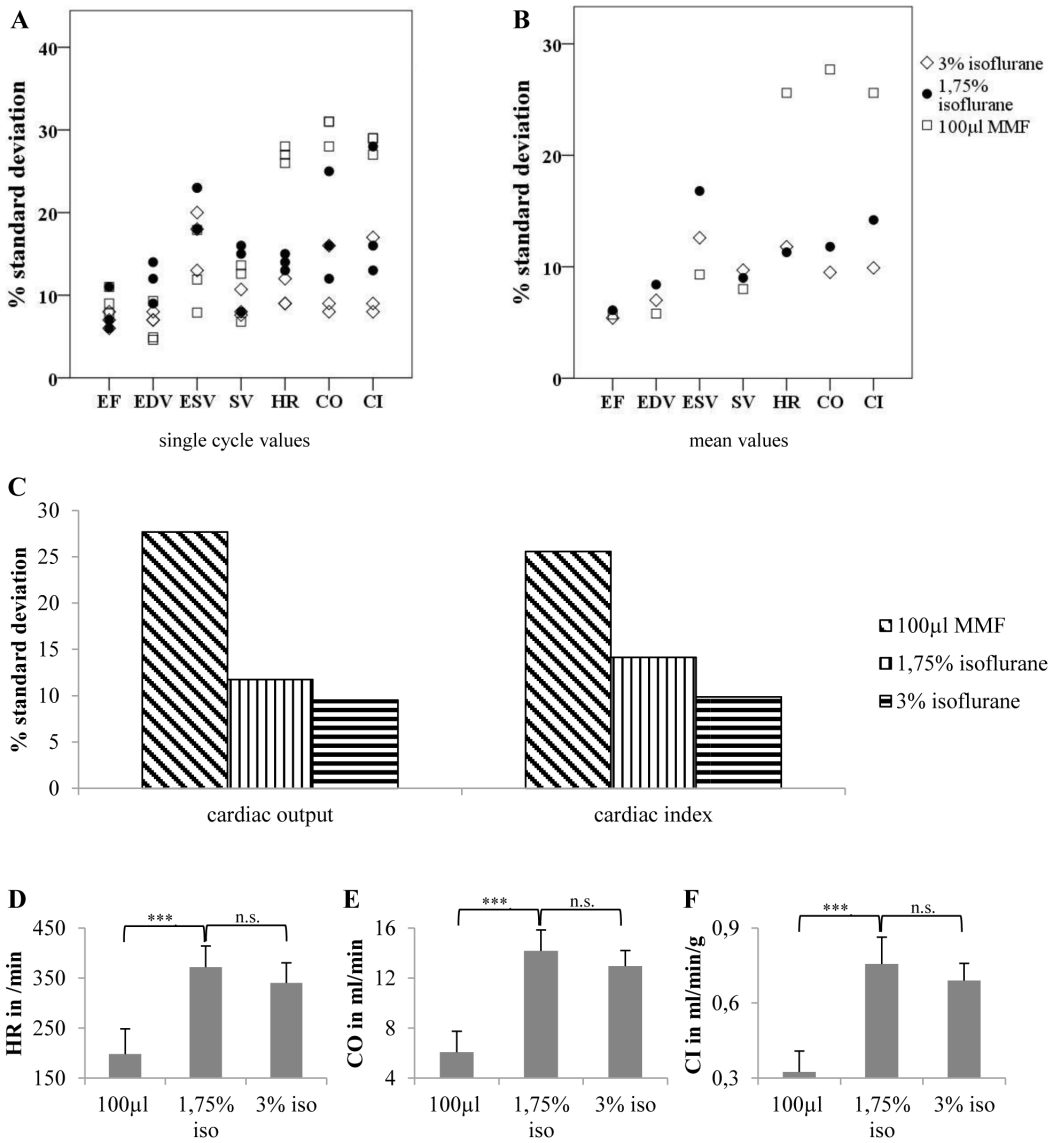
Anesthesia with isoflurane reduces variability of data. Compared to MMF, isoflurane is able to reduce the variability of the results. Additionally, isoflurane at higher concentrations than commonly used might further reduce the variability of heart rate and heart rate dependent values such as cardiac output and cardiac index. However, compared with lower isoflurane concentrations (1.75%), high isoflurane concentrations (3%) show no significant cardiodepressive effects. A) Shows percentual standard deviations of single cycle values, and B) of mean values (average of 3 consecutive cycles). (C) Shows percentual standard deviations for cardiac output and cardiac index obtained with either MMF, 1.75% isoflurane or 3% isoflurane. D) Shows heart rate, E) cardiac output and F) cardiac index under different anesthetic regimens. EF = LVEF, EDV = end diastolic volume, ESV = end systolic volume, SV = stroke volume, HR = heart rate, CO = cardiac output, CI = cardiac index. 100 µl MMF: n = 6, 1.75% isoflurane and 3% isoflurane: n = 7. ***: p<0.001.

Data regarding effects of MMF on myocardial contraction and heart rate are scarce compared to data on ketamine/xylazine or isoflurane [22]. Thus, we concentrated on cardiodepressive effects and compared ventricular volumes, cardiac function parameters and heart rates of healthy male BALB/c mice with respect to different anesthetic regimens (MMF, 1.75% isoflurane and 3% isoflurane). As shown in table 3, anesthesia with MMF results in a distinct depression of cardiac contractility (20% relative reduction of LVEF) and a dramatic deterioration of heart rate (relative reduction of 42–47%, figure 3D) and heart rate-dependent parameters such as cardiac output (relative reduction of 53–57%, figure 3E) and cardiac index (relative reduction of 60–63%, figure 3F). No differences were seen between the 1.75% and 3% isoflurane group (figure 2D and figure 3D–F), indicating that higher concentrations of isoflurane have no impact on cardiac function.

**Table 3:** Left ventricular function parameters in healthy mice under different anesthetic regimen.

	Units	1.75%	3%	MMF	RR (MMF vs. 1.75%)	RR (MMF vs. 3%)
EDV	µl	54.2	55.0	55.5	−2%	−1%
ESV	µl	16.6***	16.7***	24.6	−48%	−48%
SV	µl	37.6*	38.3*	26.5	29%	31%
LVEF	%	69.4***	69.7**	55.7	20%	20%
HR	/min	372***	340***	198	47%	42%
CO	ml/min	14.2***	13.0***	6.1	57%	53%
CI	ml/min/g	0.76***	0.69***	0.28	63%	60%

*1.75% = anesthesia with 1.75% isoflurane; 3% = 3% isoflurane. MMF = 100 µl MMF injected i.p. for maintenance of anesthesia. RR = relative reduction comparing MMF and 1.75% isoflurane and MMF and 3% isoflurane. Values are presented as mean, along with the corresponding p values (Student's t test).*

*: p<0.05,

**: p<0.01,

***: p<0.001.

*No statistical differences were observed between the 1.75% group and the 3% group.*

### Impact of the anesthetic regimen on the variability of the results

Next, we studied whether the anesthetic regimen MMF also has an impact on the variability of cardiac measurements. Therefore, we compared cardiac function parameters under MMF, 1.75% isoflurane (isoflurane is commonly used at concentrations of 1.25–2.25% for maintenance of anesthesia) and 3% isoflurane. As depicted in figure 3A+B, MMF resulted in very high standard deviations for heart rate and heart rate dependent values. On the one hand, variance of LVEF was similar in all groups. On the other hand, 3% isoflurane had the best influence on heart rate (especially in single cycle values, see figure 3A) and heart rate dependent values such as cardiac output and cardiac index (figure 3A–C and E+F). This indicates that high isoflurane concentrations might be preferable for assessment of cardiac output and cardiac index.

### Impact of calculating an average LVEF value on the variability of the results

**Figure 4:**
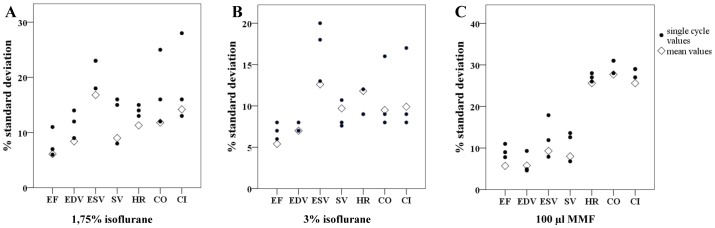
Calculation of an average LVEF value reduces variability of data independently from the anesthetic regimen. For evaluation of cardiac function parameters, 3 consecutive cycles were measured and averaged. Mean values showed a reduced variability compared to values obtained by single cycle measurements. A) Shows single cycle and mean values for the group anesthetized with 1.75% isoflurane, B) for the group anesthetized with 3% isoflurane and C) for the group anesthetized with 100 µl MMF. EF = LVEF, EDV = end diastolic volume, ESV = end systolic volume, SV = stroke volume, HR = heart rate, CO = cardiac output, CI = cardiac index. 100 µl MMF: n = 6, 1.75% isoflurane and 3% isoflurane: n = 7.

In small animal echocardiography, multiple cycle measurements are commonly used to calculate an average value for LVEF in order to reduce variability of the results [10]. In most MRI studies however, cine imaging results in the calculation of only one single LVEF value [22]–[24]. In order to investigate the effect of calculating an average LVEF value on the variability of the results, we analyzed the variability of 3 different heart cycles and compared it with the mean values (average of 3 consecutive cycles). As shown in figure 4, the mean values consisting of 3 different measurements showed lower interindividual differences for all cardiac function parameters than single cycle measurements. This effect was independent from the anesthetic regimen used. Compared to the highest standard deviation of single cycle measurements, the standard deviation of LVEF was reduced by ∼45% in the 1.75% isoflurane group, ∼38% in the 3% isoflurane group and by ∼45% in the 100 µl MMF group, indicating that acquisition of multiple cine images could be a powerful tool to reduce variability on the 1.5 T MRI device.

## Discussion

Many techniques have been implemented in the past to measure cardiac function in small animals. Pressure-volume loops can be obtained via Millar tip catheterization, but repetitive measurements are not possible in this setting. Echocardiography using clinical echo devices is often used in animal models, but provides a weak spatial resolution and is known to have a high inter- and intra-observer variability [25]. Dedicated small animal echocardiographic devices (e.g. VisualSonics Vevo2100) are very cost-intensive. Finally, 9.4 T MRI scanners dedicated to small animals have been shown to have high spatial and temporal resolutions [11], [26], [27], but are not broadly available.

In our pilot study we show that clinical 1.5 T MRI driven evaluation of left ventricular function in mice with mild cardiomyopathy is feasible, although some limitations and lessons from small animal echocardiography need to be considered.

As our data show, the variability of values obtained using a clinical 1.5 T MRI device is higher than in studies using dedicated small animal MRI or echocardiographic devices. This limitation needs to be considered regarding future study designs, as group sizes need to be increased in order to identify significant changes of left ventricular function parameters.

In order to find possible solutions for the high variability and an explanation for the depressed cardiac function of healthy controls, we verified our 1.5 T MRI results with a dedicated small animal echocardiographic device. As demonstrated above, calculation of an average LVEF as known from small animal echocardiography [10] could reduce the scattering of the values gained from 1.5 T MRI devices. Thereby, a relative reduction of standard deviation of up to 38–45% could be achieved.

Throughout literature, many anesthetic regimen including thiopental [28], 222-tribromoethanol [23], isoflurane at different concentrations (usually 1.25%–2.25%) [17], [22], [29] and ketamine/xylazine [22] have been used for evaluation of cardiac function in mice. In this article, we show that the anesthetic regimen may not only have distinct impact on cardiac function but also on the variability of the results. We showed that MMF has a marked effect on cardiac function (20% reduction of LVEF, 40% reduction of heart rate and 60% reduction of cardiac index compared to isoflurane), which makes this regimen impractical for evaluation of cardiac function. Furthermore we found, that MMF has a distinct impact on the variability of heart rate-dependent values. In contrary, higher concentrations of isoflurane than commonly used [10], [11], [17] proved to have a beneficial effect on heart rate variability.

Nevertheless, our study has some limitations. First, we only assessed healthy controls under the mentioned anesthetic regimens. Therefore, we are not able to predict the effects of the anesthetics in mice with severe cardiomyopathies. Eventually, higher isoflurane concentrations might show a distinct cardiodepressive effect in these mice leading to higher dropout rates. Second, the parasternal short axis M-Mode we used to assess cardiac function parameters is not as accurate as e.g. the LV-trace mode, which much more considers regional wall motion abnormalities.

To sum up, evaluation of LVEF changes in virus-induced cardiomyopathy with a clinical 1.5 Tesla MRI is feasible for serial monitoring of cardiac function and validation for novel therapeutic strategies. Calculation of an averaged LVEF value reduces variability. Furthermore, choice of the anesthetic regimen has essential influence on the results of cardiac measurements.
